# Towards a National System-Level Intervention: Characterization of Burnout Among Trainees of Saudi Postgraduate Healthcare Professions Programs

**DOI:** 10.3390/healthcare13050473

**Published:** 2025-02-21

**Authors:** Saud Alomar, Fahad D. Alosaimi, Maher Faden, Sami A. Alhaider, Basim S. Alsaywid, Ziad Nakshabandi, Nehal Khamis

**Affiliations:** 1 Child Health Excellence Center, King Abdullah bin Abdulaziz University Hospital, Princess Nourah Bint Abdulrahman University, Riyadh 11671, Saudi Arabia; drpeds2010@gmail.com; 2 Department of Psychiatry, College of Medicine, King Saud University, Riyadh 11362, Saudi Arabia; 3 Children’s Health Department, King Abdullah bin Abdulaziz University Hospital, Princess Nourah Bint Abdulrahman University, Riyadh 11671, Saudi Arabia; maherfaden@gmail.com; 4 Pulmonary Section, Pediatric Department, King Faisal Specialist Hospital and Research Center, Riyadh 11211, Saudi Arabia; sami.alhaider@gmail.com; 5 Education and Research Skills Directory, Saudi National Institute of Health, Riyadh 12382, Saudi Arabia; balsaywid@snih.gov.sa; 6 Pediatric Urology, Urology Section, Department of Surgery, King Saud University, Medical City, King Saud University, Riyadh 11362, Saudi Arabia; 7 National Center for Health Workforce Planning, Saudi Commission for Health Specialties, Riyadh 11614, Saudi Arabia; z.nakshabandi@scfhs.org.sa; 8 Department of Medicine, Division of General Internal Medicine, School of Medicine, Johns Hopkins University, Baltimore, MD 21205, USA; 9 Department of Advanced Studies in Education, Master of Education in Health Professions Program, Johns Hopkins University, Baltimore, MD 21205, USA

**Keywords:** trainee, physicians, burnout, harassment, discrimination, obesity, stress, depression, health profession

## Abstract

Background/Objectives: High levels of burnout among healthcare professionals and trainees represent a global problem with identified profound impacts. The collection of national data for better characterization of this problem can guide more needs-sensitive targeted interventions. We aimed to identify the prevalence of burnout, the associated factors, and their impacts among trainees of Saudi postgraduate healthcare professions training programs. Methods: We conducted an anonymous, cross-sectional survey of 11,500 Saudi Commission for Health Specialties trainees from February to May 2019. The survey included items for socio-demographic data, physical health, and work-related items. We used validated instruments to measure burnout (Maslach Burnout Inventory), stress (Perceived Stress Scale), and depression (Patient Health Questionnaire-9). Results: A total of 6606 postgraduate trainees from different healthcare professions responded (mean age of 28.8 ± 3 years). Fifty-six percent reported burnout symptoms. Burnout was lower among female trainees (aOR, 0.73; 95% CI, 0.65–0.82) and higher in trainees working ≥40 h/week (aOR, 1.19; 95% CI, 1.03–1.37) and doing ≥six on-call shifts/month (aOR, 1.18; 95% CI, 1.03–1.37). Harassment and discrimination increased the risk of burnout by 57% and 60% (aOR = 1.57, 95% CI: 1.36–1.80 and aOR, 1.60; 95% CI, 1.38–1.86), respectively. Burnout trainees had 3.57 adjusted odds to report major depression (95% CI 3.11–4.09), were 1.25 times more likely to report major stress (95% CI 1.36–1.80), and were 1.8 times more likely to complain of sleep disorders (95% CI 1.60–2.04). Conclusion: This study identified several personal and work-related risk factors and impacts of burnout among our postgraduate trainees. The findings were helpful in guiding the expansion of the national Da’em well-being and prevention of burnout program efforts to a targeted system-level intervention.

## 1. Introduction

Clinician burnout is a threat to healthcare providers, patients, and the entire healthcare system [1,2]. The World Health Organization (WHO) considers burnout as an occupational phenomenon associated with employment rather than a mental health diagnosis. Accordingly, WHO has updated its definition of burnout to ‘a syndrome resulting from chronic workplace stress that has not been successfully managed’ [3].

Healthcare providers’ burnout is a significant global problem. For example, it has been estimated that more than half of physicians in the United States have at least one symptom of professional burnout [4]. The 2024 Medscape National Physician Burnout and Suicide Report revealed a prevalence of 49% [5]. The magnitude of the problem is also expressed in the high rates of burnout reported among nurses. In a study by Kelly et al. [6], it was found that 54% of the sampled nurses were suffering from burnout, which had reached high levels in 28% of them.

Among healthcare workers, postgraduate trainees seem to be more vulnerable to burnout [7]. Several factors subject postgraduate trainees to tremendous amounts of pressure. For instance, career opportunities and duties that are very competitive and demanding make postgraduate training a very stressful career phase for healthcare professionals. In addition, trainees are subjected to continuous clinical and moral challenges; knowledge, technical, and non-technical skills assessments; and variable expectations [7,8].

In a meta-analysis study, it was found that 50% of trainee physicians were experiencing burnout symptoms [9]. While research on burnout is beginning to emerge globally in addition to the Western data, the understanding of burnout is likely shaped by cultural contexts and organizational behaviors in various settings, as suggested by Hofstede’s theory on cultural dimensions impacting workplace burnout [10]. We propose that the incidence of burnout may be more pronounced among healthcare professionals in Arab countries, given that their healthcare systems and funding models are either heavily strained or rapidly evolving to address changing disease patterns, all within a distinct cultural framework for healthcare delivery compared to Western systems [11]. Nationally, only a few studies have investigated burnout among postgraduate healthcare trainees. In one study, 25% of the responding radiology residents had high burnout, and more than half of them scored high on emotional exhaustion [12]. Alfaleh HM et al., 2017, also reported more significant levels of burnout among family and internal medicine residents [13,14]. In their ‘Progressive Model for Quality Benchmarks of Trainees’ Satisfaction in Medical Education’ [15], the authors reported that almost two-thirds of the responding residents agreed that they ‘feel that they experience a significant rate of burnout that has key impacts on their professional conduct and lives’.

There are many factors that can contribute to professional burnout. Given the complexity of burnout as a syndrome, these factors have been integrated into multiple burnout etiological explanatory models, for example, the job demand-resources model [16], the effort-reward imbalance model [17], and the organizational injustice model [18]. The imbalance between job demand and resources plays a significant role in developing burnout among healthcare professionals [13,19]. This applies to health professions trainees as well.

Job demands include all aspects of workload such as role ambiguity, role conflict, task difficulty, administrative burden, time pressure, overtime hours, distractors, travel time, and others. On the other hand, job resources may include personal resources and resilience, self-efficacy, relationships and social support, administrative support, meaning at work, job control, autonomy, and others [20,21].

Burnout has many serious physical and professional consequences. Healthcare professionals and trainees suffering from burnout might be at higher risk for developing cardiovascular diseases, hypercholesterolemia, type 2 diabetes, musculoskeletal pain, and headaches [22]. They are also more prone to depression and higher rates of suicidal attempts [23,24]. Moreover, job dissatisfaction, specialty change, and career shift are reported among burnt-out health professions trainees [20,21,22,25].

Published studies investigating residents’ burnout are limited in many instances to one-specialty candidates [9]. Multi-specialty/multi-occupation burnout studies, on the other hand, still also have the limitation of using a retrospective systematic review methodology that includes studies using different burnout definitions and assessment tools and diverse targeted populations. This hinders the external validity of the results reached. The aim of the current study is to characterize the problem of burnout among the health professions postgraduate trainees enrolled in the Saudi Commissions for Health Specialties (SCFHS) training programs. Our objectives include estimating the prevalence of burnout among the different health profession education trainees using validated tools and identifying the associated risk factors and impacts of burnout.

To our knowledge, this is the first national and regional study to investigate the burnout problem among the large scale of all postgrad health professions training programs. Hopefully, the results of this research will inform and guide the efforts toward expanding the SCFHS ‘Da’em’ residents’ wellness support program to incorporate and implement needs-sensitive and evidence-based wellness-enhancing, burnout preventive and curative strategies. The team’s ultimate goal is to translate these into policies and procedures targeting all involved stakeholders in one systems-based human factor model [25]. The purpose of our study is to identify the prevalence of burnout, the associated factors, and their impacts among trainees of Saudi postgraduate healthcare professions training programs.

## 2. Materials and Methods

### 2.1. Study Design and Population

We conducted a national, anonymous cross-sectional analytical study between 1 February and 30 May, 2019 as part of the national wellbeing program under SCFHS. The study used an online self-administered survey that targeted all 11,500 trainees enrolled by that time in all the SCFHS-accredited training programs, which are located only in Saudi Arabia, Bahrain, and the United Arab of Emirates (UAE). All healthcare profession trainees were eligible to participate in the study, including physicians (medical and surgical), nurses, pharmacists, and applied health professionals. Email invitations were sent to all trainees, and the survey was accessed through trainees’ SCFHS accounts. In addition, the survey was presented during the national wellbeing program ‘Da’em’ awareness campaign conducted in the four training regions in Saudi Arabia (Central, Eastern, Western, and Northern). The survey was open for data collection for three months, with weekly reminders sent over the three months. In addition, program directors and chief residents were contacted to remind the trainees to respond to the survey. Utilizing the Raosoft R software (Raosoft Inc. (2004), Seattle, WA, USA) for sample size calculation and based on the estimated parameters of the study population, the required sample size is much lower than what we have achieved in this study utilizing census sampling (6606 participants). For a total target population of 11,500 trainees, with a 95% confidence level and a 5% margin of error, the minimum sample size was estimated to be 372.

Before starting the survey, we requested that all trainees sign an online participation consent after informing them of the study’s objectives, confidentiality of their responses, and their right to refuse or agree to participate without any consequences.

### 2.2. Questionnaire

We used validated instruments to measure burnout, stress, and depression. For the broader questionnaire, face and content validity methods were used. A multidisciplinary group of experts including psychiatrists, psychologists, and researchers designed the questionnaire (Table S1). The questionnaire items were reached by consensus of the team members after reviewing the relevant literature regarding the diagnosis of, factors associated with, and impacts of burnout [26,27,28,29,30].

The survey included five sections:

1. Demographic data: The 16 items in the first section covered sociodemographic data (gender, age, marital status, having children, monthly income, training region), training program, and work-related demands (specialty, stage of training, working hours, travel time to work, number of on-call shifts/month, and exposure to harassment or discrimination at the workplace). This is in addition to non-work-related physical health items including level of daily exercise, BMI, and presence of a chronic disease.
2. Stress level: The second section in the survey explored the level of stress among the trainees. The Perceived Stress Scale (PSS) was used [31]. This is rated with a 5-point Likert scale (0 never, 1 almost never, 2 once in a while, 3 often, 4 very often), with higher scores reflecting higher levels of perceived stress [32,33,34].
3. Burnout: The third section assessed burnout by using the MBI-HSS (MP) [35]. The MBI-HSS has reported validity evidence for use among health professionals [36]. We obtained approval from Mind Garden (http://www.mindgarden.com, accessed on 16 February 2025) to use the full version of MBI -22 items (license number #24086). The MBI’s 22 items consist of three subscales: emotional exhaustion (ranging from 0 to 54 points), depersonalization (ranging from 0 to 30 points), and reduced personal accomplishment (ranging from 0 to 48 points). Each subscale item is rated using a 7-point Likert scale (0 = never to 7 = everyday). Emotional exhaustion questions screen for feelings of being emotionally overextended and exhausted at work. Depersonalization questions discover the presence of unfeeling and impersonal responses towards patients. Personal accomplishment questions measure feelings of competence and achievement at work. Each subscale is designed to measure three levels (low, medium, and high). Burnout, in this study, was defined as a high score on the emotional exhaustion and/or depersonalization subscales [37].
4. Depression: The fourth section evaluated the presence of depression. The PHQ-9 depression scale was used to assess depressive burden in trainees [38,39]. The PHQ-9 is a self-administered version of the PRIMEMD diagnostic instrument for common mental disorders [38]. The PHQ-9 score can range from 0 to 27 since each of the nine items can be scored from 0 (not at all) to 3 (nearly every day). Total scores ranging from 1 to 4 are considered normal levels of depression, 5–9 are considered mild, 10–14 are moderate, 15–19 are moderately severe, and 20–27 are severe depressive symptoms [38]. For the purpose of studying the impact of burnout, we defined major depression as a PHQ-9 score of 10 or more [39].
5. Burnout’s impact: The fifth section in the survey included items to explore other potential impacts of burnout reported in the literature in addition to depression [40], namely, dropping from the training program, considering change in specialty, job satisfaction, and sleep disorders.

The final questionnaire content and sequence were reached after piloting the first version on 40 trainees from different specialties to assess reliability, the time needed for survey completion, and any feedback from the respondents about the content and clarity of the items.

### 2.3. Statistical Analysis

We performed our statistical analysis using IBM SPSS Statistics for Windows, version 23.0 (IBM Corp., Armonk, NY, USA). Saudi healthcare trainees’ characteristics were compared between the exposure (burnout) and control (no burnout) groups using univariate analyses. To test for statistical significance, we used Student’s *t*-test for normally distributed continuous variables (or the Mann–Whitney test for continuous non-parametric variables). For categorical variables, the chi-squared test (or Fisher’s exact test when appropriate) was used. To study the association between the independent variables and the dependent variable (burnout), crude and adjusted odds ratios were calculated. We built a backward stepwise logistic regression model according to the likelihood ratio test to identify the significant independent risk variables associated with burnout. Significant variables that had a *p*-value < 0.25 in univariate analysis were included in this model. However, only variables with a *p*-value < 0.05 were included in the final model. To study burnout’s impact, we calculated crude and adjusted odds ratios. We included in the model burnout and significant risk factors from the previous regression analysis in the final model.

## 3. Results

### 3.1. Response Rate

A total of 6606 trainees completed the survey (N = 6606/11,500, 57.8%). Of the responding 6606, only four trainees did not complete the MBI-HSS, and all trainees completed the PHQ-9 and the PSS.

### 3.2. Characteristics of the Respondents

The characteristics of the healthcare profession trainees who completed the survey are shown in Table 1. The respondents included 3687 (56%) males and 2916 (44%) females, with a mean (SD) age of 28.8 (3.00) years, and almost half of the responders were single (46%). Medical specialties physicians represented most of the responders (53%), followed by surgical specialties physicians (30%). There was good representation of both junior level (≤2 years in training) and senior level trainees (>2 years in training).

The response rate, based on total trainees on each region, ranged between 41% and 69%. The highest response rate was in the east region (69%), and the lowest was in the Bahrain region (41%). However, both central and west regions represent 73% of the total trainees in Saudi Arabia (Table 2).

### 3.3. Prevalence of Burnout

The overall prevalence of burnout among healthcare profession trainees was 56% (3704 trainees). The highest prevalence of burnout, when categorized by healthcare occupation, was among surgical trainees and applied health services trainees (60% for each). The prevalence of burnout among the other healthcare professions trainees ranged from 50 to 55%. The prevalence of burnout was highest among the western region trainees (60%), while the lowest prevalence was found in the northern region (47%). There was no significant difference in the prevalence between male and female trainees, which were 55% and 57%, respectively (Table 1).

### 3.4. Factors Associated with Burnout in the Univariate Analysis

In the univariate analysis (Table 3), we did not find a significant association between gender, age, marital status, having children, or level of training variables and the presence of burnout. Among the physical health risk factors, healthcare trainees with chronic diseases (crude OR 1.55, 95% CI: 1.32–1.82, *p*-value < 0.0001), those rarely performing exercise (crude OR 1.65, 95% CI: 1.26–2.15, *p*-value < 0.0001), and the overweight/obese (crude OR 1.21, 95% CI: 1.07–1.37, *p*-value = 0.002) were at higher odds of reporting burnout. Physician trainees in surgical training programs (crude OR 1.29, 95% CI: 1.15–1.44, *p*-value < 0.0001) and trainees in eastern (crude OR 1.18, 95% CI: 1.02–1.37, *p*-value = 0.02) and western regions (crude OR 1.15, 95% CI: 1.02–1.29, *p*-value = 0.01) were more likely to report burnout. Trainees whose monthly income was in the range of 15,000–20,000 Saudi Riyals (about $4000–5300) in comparison to trainees whose monthly income was more than 20,000 Saudi Riyals (crude OR 1.19, 95% CI: 1.02–1.38, *p*-value = 0.02), those working more than 40 h per week, and those doing more than six on-call shifts per month were at higher risk of burnout. Trainees exposed to harassment (crude OR 2.54, 95% CI: 2.26–2.85, *p*-value < 0.0001) and discrimination (crude OR 2.48, 95% CI: 2.19–2.81, *p*-value < 0.0001) had higher odds of having burnout as well. Trainees with higher scores on the PSS had higher odds of burnout (crude OR 1.26, 95% CI: 1.24–2.81, *p*-value < 0.0001).

### 3.5. Multivariable Model for Risk Factors That Might Predict Burnout

The logistic regression model was conducted in ten steps by backward stepwise analysis. Seventeen variables were included. The sequence of variables that were removed because they did not reach statistical significance at each stage was as follows: age, marital status, having children, having a chronic disease, BMI, training region, level of training, and monthly income. After adjusting for multiple variables in the logistic regression analysis, the following findings were associated with burnout: gender (male reference) (adjusted OR = 0.73, 95% CI: 0.65–0.82, *p*-value < 0.0001), working more than 40 h per weeks (adjusted OR = 1.19, 95% CI: 1.03–1.37, *p*-value = 0.017), working more than six on-call shifts per month (adjusted OR = 1.18, 95% CI: 1.03–1.37, *p*-value = 0.017), Perceived Stress Scale (adjusted OR = 1.25, 95% CI: 1.23–1.27, *p*-value = 0.017), harassment (adjusted OR = 1.57, 95% CI: 1.36–1.80, *p*-value < 0.0001), and discrimination (adjusted OR = 1.60, 95% CI: 1.38–1.86, *p*-value < 0.0001), Table 4.

### 3.6. Impact of Burnout on Trainees

Among respondents, 40% (2645/6606) reported positive symptoms for major depression on the PHQ-9 screening test. In the survey section measuring satisfaction with the training program, around 34% considered changing or dropping out from their training program, 34% considered quitting their specialty, and 4% had already dropped. Forty percent of trainees reported sleep disorders: insomnia, sleep apnea, restless leg syndrome, and sleepwalking (Table 5).

### 3.7. Multivariable Model of Impact of Burnout

In a multivariable model studying the impact of burnout among trainees, we adjusted for sex, age, relationship status, healthcare profession, level of training, training region, having children, having a chronic disease, BMI, doing exercise, travel time to work, number of on-call shifts per month, monthly income, and exposure to harassment or discrimination (Table 4). Overall, postgraduate trainees of healthcare professions experiencing burnout were three and half times more likely to have major depression (aOR, 3.57, 95% CI: 3.11–4.09; *p*-value < 0.0001). Burnout also correlated with increasing odds of dropping from the training program (aOR, 1.36; 95% CI: 1.05–1.84; *p* = 0.02), considering changing specialty (aOR, 2.29; 95% CI: 2.01–2.61; *p* < 0.000), and considering quitting specialty (aOR, 2.23; 95% CI: 1.95–2.54; *p* < 0.0001). Trainees with burnout had five times greater ‘very little satisfaction’ with their current job (aOR, 5.13; 95% CI: 3.42–7.69; *p* < 0.0001). Healthcare trainees with burnout are at a 1.8 higher risk of sleep disorders in comparison with trainees without burnout (aOR 1.81; 95% CI: 1.60–2.04, *p* < 0.0001) (See Table 5).

## 4. Discussion

Among healthcare profession trainees enrolled in the SCFHS training programs across Saudi Arabia and training sites at Bahrain and UAE, symptoms of burnout were reported by 56% of the 6606 respondents. The fully adjusted model of analysis in our study indicated that risk factors of burnout are male gender, working ≥40 h/week, doing ≥six on-call shifts/month, and harassment and discrimination. Moreover, the impact of burnout includes major depression, major stress, and sleep disorders. The specialty with the highest reported residents’ burnout in the current study was surgery with a prevalence of 60%. This is very close to the 58% percent reported by Low et al. [41], who performed a meta-analysis of the prevalence of burnout among residents. Our results are also consistent with earlier national findings. In AlQahtani et al.’s [42] survey involving 330 general surgery residents in SCFHS training programs, 46.2% of the respondents reported moderate to high levels of burnout (30% had high emotional exhaustion, 39% showed high depersonalization, and 61.4% expressed a low sense of personal accomplishment). In addition, Aldrees et al. [43] found that approximately half of the plastic surgery trainees in Saudi Arabia have signs of professional burnout.

Although our study was conducted before the COVID-19 crisis, we believe the findings remain relevant, as some international data suggest that burnout levels are gradually returning to pre-COVID levels. The Medscape Report 2024 among US doctors indicated less burnout (49%) in 2024 compared to 2023, after steady climbs through the worst COVID years from 42% in 2018 to 53% in 2023 [5]. During the COVID era, the significant influx of patients, along with the emotional strain of witnessing serious illnesses and deaths, was a major contributor to physician burnout. Furthermore, the rapid adoption of new technologies and changes in healthcare delivery to facilitate remote consultations and telemedicine have added to the strain on physicians’ work-life balance. The initial lack of personal protective equipment (PPE) and the heightened risk of virus exposure also contributed to the stress faced by healthcare workers. To tackle these challenges, it is essential to implement comprehensive support systems that include mental health resources, effective communication, and strategies for managing workloads to alleviate the effects of COVID-19 on physician burnout and maintain the healthcare workforce [44].

The high prevalence rates of burnout reported in our study among trainees of certain specialties—surgery, ICU, ER, and anesthesia—have been attributed in the literature to the unique nature of these specialties’ professional tasks [19]. Another interesting explanation is derived from the fact that the prevalence of burnout among these specialty trainees is no different than the burnout prevalence among these specialties’ practitioners [45]. The influence of the specialty and the role model effect on the residents is an area for investigation in future research.

Analysis of the balance between work demands and work support resources in training programs and institutes is quite important for the prevention of burnout. The traditional system of postgraduate training might present potential academic pressure, especially with the high weight of summative assessment and less emphasis on formative feedback. The current trend of moving towards entrustment with gradual progressive autonomy granted to trainees might enhance trainees’ confidence and improve performance [46] and might, as such, reduce the levels of academic stress and, accordingly, burnout. Further research in this area is needed.

Regarding occupations other than physicians, the highest prevalence rates reported in our current study are among applied health providers and pharmacists. These findings need to be interpreted cautiously in view of these professions’ low sample representation (1% and 0.5%, respectively). Yet, our results are consistent with findings from the literature reporting burnout rates of 41% among pharmacists [47], high rates of emotional exhaustion (58%), negative feelings about work and clients (94%), and scarce sense of personal accomplishment (1%) among US applied health professionals (physiotherapists and occupational therapists) [48] and a self-reported burnout level (emotional exhaustion > 27 and/or depersonalization > 10) of 67.4% among applied health professionals in Singapore [49].

In our study, we explored sociodemographic, physical health, and work-related risk factors that could lead to an increase in burnout among Saudi healthcare postgraduate programs’ trainees. Among sociodemographic data, we found no differences in the prevalence of burnout based on gender, as burnout was 55% in males and 57% in females. However, in logistic regression, females were 27% less likely to report burnout in comparison to males. Most of the studies reporting burnout based on gender found that females are at higher risk of burnout [50,51,52]. We attributed our finding that females are at less risk of burnout to the state of equality in training rights and social support to female health professionals in Saudi Arabia. We found no association between burnout and age or marital status in both crude and adjusted analysis. These results are consistent with those of Zhou et al. [36], as well as the findings of the Saudi national survey of burnout among general surgery residents [42].

A total of 11% of our respondents reported chronic disease, and almost more than half had burnout (55%). The crude OR is 1.55, *p*-value < 0.0001, but when we adjusted for other risk factors, chronic disease was not a predictor of burnout. However, trainees with chronic diseases should be encouraged and supported at all stakeholder levels to take care of themselves and given opportunities to attend their regular appointments with their physicians.

Overweight and obesity status increased the odds of burnout by 20%, but not in the adjusted model. The association between overweight and obesity status and burnout needs further longitudinal investigation. It is not clear whether overweight and obesity status results in stress or burnout or vice versa. For instance, patients experiencing occupational stress and burnout are more vulnerable to emotional and uncontrolled eating [53], and the prolonged subjection to stress hormones might lead to abdominal obesity and metabolic syndrome [54].

Regular exercising is a protective factor against burnout [42,55,56]. In our study, trainees who rarely exercised were at greater risk of burnout by 65% (crude OR. 1.65, 95% CI: 1.26–2.16, *p*-value < 0.001). Training institutes are encouraged to emphasize the importance of physical activity on a trainee’s well-being and its great impact on burnout and stress reduction. This can be done through awareness campaigns, facilitating physical activities in the workplace, collaboration with fitness clubs, and supplying discount coupons for gym subscriptions.

Extended-duration shifts (>80 h per week) and spending more nights on call result in a decrease in sleeping and rest hours [57,58,59]. These are sources of both physical and psychological stress that can lead to burnout over time [60]. In the current study, we found a statistically significant association between working more than 40 h/week and doing more than six on-call shifts per month and burnout. In a survey including 118 residents and interns, the prevalence of burnout was 31% higher among trainees who worked >80 h/week than those who worked <80 h/week [61]. The cut-off point that we used in the current study was 40 h/week; however, Hameed et al. [62], in their multi-institutional study of residents’ burnout and duty hours in Saudi Arabia, found that half of their responding residents worked 60–79 h/week. Thirty percent reported working ≥80 h/week, and more than two-thirds worked 24–28 h when on call. Monitoring the implementation of national policies that regulate residents’ working hours and on-call shifts per week is highly recommended. Securing 6 to 8 h/day of sleep and adequate days off protect against healthcare provider burnout [63].

In our research, almost one quarter of the respondents faced a form of harassment or discrimination, and 72% of them reported significant rates of burnout symptoms. These findings are worth further investigation. Harassment and discrimination are noted to be key predictors of burnout among physicians and healthcare trainees [64,65]. Arora and Blanchard [26] analyzed the association between bias as an expression of discrimination and burnout, and we totally agree with their conclusion that neither previous studies nor ours can confidently assume a causal relationship. Burnout might have simply unmasked the feelings of bias/discrimination that our residents have been subjected to. As such, developing strategies to mitigate both implicit and explicit forms of discrimination and burnout are of utmost importance [28,66].

Consequences of burnout like lower job satisfaction, absenteeism, and quitting the training program are commonly reported in the literature [67,68]. Burnout correlated, in our study, with increasing odds of dropping from the training program, considering changing or quitting one’s specialty, and five times higher ratings of ‘very little satisfaction’ with the current job. Previous studies among Saudi residents have shown similar results [69]. The findings from Bin Dahmash et al. [70] matched the previously reported significant association between emotional intelligence, job satisfaction, and burnout [71,72]. Enhancing emotional intelligence at the workplace is a potential protective measure against burnout that is worth investigation.

Major depression symptoms were reported by 40% of the Saudi healthcare postgraduate programs’ trainees. In addition, we found a strong relationship between burnout and major depressive symptoms, as trainees with burnout reported around four times greater depressive symptoms. Mata et al. [73] reported in their meta-analysis that the prevalence of major depressive symptoms ranged between 20 and 40% among residents. This matches our findings, as well as the strong link between burnout and depression reported in previous publications [74,75]. This serious finding mandates taking effective preventive, early detection, and management measures at both personal and organizational levels.

Our study has the strengths of targeting trainees from all health professions enrolled in the SCFHS programs in three countries with a large overall sample size of 6606 participants. The study has used validated tools of evaluation and investigated different aspects of the burnout syndrome, including prevention, contributing factors, and possible consequences. In addition, we used multi-variate analysis to better identify the associated risk factors. Potential limitations of the current study include the following: (1) Most respondents were physician residents enrolled in training programs inside Saudi Arabia with far less representation of trainees from other healthcare professions and those from Bahrain and UAE. This can limit the external validity and ability to generalize the results to trainees from different health professions. (2) The cross-sectional design of the current study makes it difficult to determine the cause and effect and direction of the relationship. Future longitudinal studies with baseline measurements can better assess the time-related variables that contribute to the development of burnout. (3) Like in other studies, it is hard to investigate all factors relevant to the complicated concept of burnout and its occurrence; therefore, there are work-demand, work-engagement, and resources factors that were not investigated in the current study. (4) Only face and content validity methods were employed, both of which are subjective and not quantifiable, potentially leading to certain limitations. Although the mental health assessment scales have been validated, the overall questionnaire would benefit from further validation efforts to improve its reliability and robustness.

The findings of the current study as well as many focused group discussions with the various stakeholders have been invaluable resources in reaching a Da’em system-level intervention framework for trainees’ well-being in the year 2022. This framework considers a real-life analysis of the contributions of each of the four major stakeholder groups (trainees, training program, training center, and accrediting body) to job demands, job resources, and engagement with the provision of targeted interventions at the various levels of the system.

The framework was developed by a multi-disciplinary team and follows recent approaches to professional well-being, including the National Academies of Sciences, Engineering, and Medicine report, “Taking action against clinicians well-being: A systems approach to professional well-being” [25], presented at the 2020 ACGME Educational Conference in San Diego, USA. The framework and preliminary data of its positive impact in combating burnout will be published in a future article.

## 5. Conclusions

Our national, cross-sectional study aimed to identify the prevalence of burnout, the associated factors, and their impacts among trainees of Saudi postgraduate healthcare professions training programs. We found burnout to be prevalent among SCFHS trainees (56%). Risk factors of burnout were male gender, working ≥40 h/week, doing ≥six on-call shifts/month, and harassment and discrimination. Moreover, the impacts of burnout included major depression, major stress, and sleep disorders. Regulating working hours and expansion of the Da’em trainees’ wellbeing program to involve all stakeholders within a system-based intervention framework are expected to ameliorate burnout impacts on trainees, patients, and healthcare. There is a need for longitudinal studies to re-evaluate changes in burnout prevalence, risk factors, and impacts, particularly in the post-COVID-19 era. Additionally, research should examine the effectiveness of specific well-being programs in addressing burnout among healthcare trainees, both nationally and internationally.

## Figures and Tables

**Table 1:** Demographic and other characteristics by burnout status among SCFHS healthcare trainees in Saudi Arabia (n = 6066).

Variables	Overall N (%) (n = 6066)	Burnout N (%) (n = 3704)	No Burnout N (%) (n = 2362)	*p*-Value
Sociodemographic				
Gender				
Male	3687 (56)	2036 (55)	1651 (45)	0.009
Female	2916 (44)	1668 (57)	1244 (43)	
Age, mean (S.D)	28.8 (3.00)	28.7 (2.7)	28.9 (3.2)	0.009
Marital status				
Single	3058 (46)	1731 (57)	1326 (43)	0.64
Married	3413 (52)	1899 (56)	1512 (44)	
Divorce	127 (2)	71 (56)	55 (44)	
Widow	4 (0)	2 (50)	2 (50)	
Having Children	2287 (35)	1252 (55)	1033 (45)	0.11
Non-work-related physical health				
Having chronic disease	719(11)	470 (66)	248 (35)	0.000
Exercise				
Rarely	3623 (55)	2212 (61)	1409 (39)	0.000
1–4 times/weeks	2742 (41)	1375 (50)	1367 (50)	
Everyday	238 (4)	115 (49)	121 (51)	
BMI				
Normal	3426 (52)	1867 (54)	1559 (46)	0.000
Underweight	1561 (24)	882 (57)	678 (43)	
Overweight/obese	1549 (24)	916 (59)	630 (41)	
Training program and work-related demands				
Occupation				
Physician medical specialty	3504 (53)	1900 (54)	1601 (47)	0.001
ICU’s ER	511 (8)	285 (56)	226 (44)	
Pharmacy specialty	29 (0.5)	16 (55)	13 (45)	
Physician Surgical specialty	1950 (30)	1179 (60)	770 (40)	
Dental	366 (6)	194(53)	172 (47)	
Nurses	185 (3)	93 (50)	92 (50)	
Allied health services	52 (1)	31 (60)	21 (40)	
Training Region				
Central	2280 (35)	1241 (54)	1039 (46)	0.006
East	1107 (17)	649 (59)	457 (41)	
West	2393 (36)	1386 (58)	1007 (42)	
North	187 (3)	88 (47)	99 (52)	
South	541 (8)	288 (53)	253 (47)	
Bahrain	56 (1)	32 (57)	24 (43)	
UEA	30 (1)	14 (47)	16 (53)	
Level of training				
Subspeciality Fellowship level	75 (1)	36 (48)	39 (52)	0.06
Junior level (1–2 years in specialty training)	3578 (54)	1964 (55)	1614 (45)	
Senior level (>2 years in specialty training)	2937 (45)	1698 (58)	1239 (42)	
Monthly income				
Less than 15,000 SR	787 (12)	439 (56)	346(44)	0.064
15,000–20,000 SR	4980 (75)	2817(57)	2146(43)	
More than 20,000 SR	824 (13)	430 (52)	391 (48)	
Working hours more than 40 h/week	5108 (77)	2981 (58)	2127 (42)	0.000
Traveling time to work > 30 min	2376 (36)	1386 (58)	982 (42)	0.003
Working more than 6 calls/month	1503 (23)	944 (63)	555 (37)	0.000
Exposure to harassment	1858 (28)	1333 (72)	525 (28)	0.000
Exposure to discrimination	1546 (23)	1117 (72)	429 (28)	0.000
Perceived Stress Scale. Mean (S.D.)	19.5 (5.9)	22.1 (5.1)	16.2 (5.3)	0.000

**Table 2:** Response rate by regions.

Region	Total Number of Trainees	Response Rate
Central	4710	2280 (48%)
West	3794	2393 (63%)
East	1593	1107 (69%)
North	300	187 (62%)
South	900	541 (60%)
UEA	69	30 (43%)
Bahrain	134	56 (41%)

**Table 3:** Univariate analysis for predicting the risk of burnout.

Variables	Crude OR	CI 95%	*p*-Value
Sociodemographic			
Gender			
Male	1 (reference)		
Female	1.08	0.98–1.19	0.09
Age, mean (S.D.)	0.98	0.97–1.00	0.086
Marital status			
Single	1 (reference)		
Married	0.96	0.87–1.06	0.44
Divorced	0.98	0.69–1.41	0.95
Widowed	0.76	0.10–5.44	0.79
Having children	0.92	0.83–1.02	0.11
Having chronic disease	1.55	1.32–1.82	0.000
Non-work-related physical health			
Exercise			
Rarely	1.65	1.26–2.15	0.00
1–4 times/weeks	1.05	0.81–1.38	0.67
Everyday	1 (reference)		
BMI			
Normal	1 (reference)		
Underweight	1.08	0.96–1.22	0.18
Overweight/obese	1.21	1.07–1.37	0.002
Training program and work-related demands			
Occupation			
Physician medical specialty	1 (reference)		
ICU’s, ER	1.06	0.88–1.28	
Pharmacy specialty	1.03	0.49–2.16	
Physician surgical specialty	1.29	1.15–1.44	
Dental	0.95	0.76–1.17	
Nurses	0.85	0.63–1.14	
Allied health services	1.24	0.72–2.17	
Training region			
Central	1 (reference)		
East	1.18	1.02–1.37	0.02
West	1.15	1.02–1.29	0.01
North	0.74	0.55–1.00	0.05
South	0.95	0.79–1.15	0.61
Bahrain	1.11	0.65–1.90	0.68
UEA	0.73	0.35–1.50	0.39
Level of training			
Fellowship level	1 (reference)		
Junior level (1–2 years in training)	1.31	0.83–2.04	0.23
Senior level (>2 years in training)	1.48	0.93–2.34	0.09
Monthly income			
Less than 15,000 SR	1.15	0.94–1.40	0.16
15,000–20,000 SR	1.19	1.02–1.38	0.02
More than 20,000 SR	1 (reference)		
Working hours more than 40 h/week	1.50	1.33–1.68	0.000
Traveling time to work > 30 min	1.16	1.04–1.28	0.004
More than 6 on-call shifts/month	1.45	1.29–1.63	0.000
Harassment	2.54	2.26–2.85	0.000
Discrimination	2.48	2.19–2.81	0.000
PSS	1.26	1.24–1.28	0.000

**Table 4:** Outcome of the binary logistic regression analysis for related risk factors and burnout in SCFHS healthcare trainees.

	Adjusted OR	CI	*p*-Value
Gender (Male reference)	0.73	0.65–0.82	0.000
Working hours more than 40 h/week	1.19	1.03–1.37	0.017
>6 on-call shifts/month	1.18	1.03–1.37	0.017
Perceived Stress Scale	1.25	1.23–1.27	0.000
Harassment	1.57	1.36–1.80	0.000
Discrimination	1.60	1.38–1.86	0.000

**Table 5:** Univariate and multivariable regression models of the impact of burnout on SCFHS healthcare trainees.

Variable	Total Respondents’ Number	Total Number with Burnout (%)	Crude OR	95% CI	*p*-Value	aOR	95% CI	*p*-Value
Major depression	6604	2645 (40)	8.07	7.16–9.13	0.000	3.57	3.11–4.09	0.000
Dropped from training program at any time since starting the training	6574	319 (4)	1.65	1.30–2.10	0.00	1.36	1.05–1.84	0.02
Considered changing specialty at least once in last month	6601	2249 (34)	3.72	3.32–4.17	0.00	2.29	2.01–2.61	0.000
Considered quitting specialty at least once in last month %	6585	2258 (34)	4.10	3.69–4.60	0.000	2.23	1.95–2.54	0.000
How satisfied are you with your current job?	6600							
0 very little		302 (4.6)	19.3	13.3–28.2	0.000	5.13	3.42–7.69	0.000
1		339 (5.1)	17.4	12.30–24.6	0.000	5.99	4.12–8.72	0.000
2		697 (10.6)	11.2	8.72–14.3	0.000	4.40	3.33–5.80	0.000
3		1623 (24.6)	6.18	5.07–7.53	0.000	2.90	2.32–3.60	0.000
4		1830 (27.7)	2.87	2.37–3.48	0.000	1.71	1.38–2.12	0.000
5		1095 (16.6)	1.57	1.28–1.94	0.000	1.19	0.94–1.50	0.14
6 very much		714 (10.8)	1 (reference)					
Sleep disorders (insomnia, sleep apnea, restless leg syndrome, and sleepwalking)	6603	2645 (40)				1.81	1.60–2.04	0.000

## Data Availability

The original contributions presented in this study are included in the article material. Further inquiries can be directed to the corresponding author.

## Supplementary Materials

The following supporting information can be downloaded at https://www.mdpi.com/article/10.3390/healthcare13050473/s1, Table S1: The study questionnaire.
