# Supplementary material for: Towards a National System-Level Intervention: Characterization of Burnout Among Trainees of Saudi Postgraduate Healthcare Professions Programs
Source: Healthcare (Basel). 2025 Feb 21;13(5):473. doi: 10.3390/healthcare13050473 (PMC11898503; doi:10.3390/healthcare13050473)
Supplement: Supplementary file 1 [file healthcare-13-00473-s001.zip › healthcare-3439190-supplementary.pdf]

**Sociodemographic Information**	
1. What is your gender?	Male/ Female
2. What is your age?	____ years
3. What is your marital status?	Single/ Married/ Divorced/ Widow
4. How many children do you have?	
5. What is your monthly income?	Less than 15,000/ 15,000 - 20,000/ 20,000 - 30,000/ More than 30,000
**Training program, and work-related demands Information**	
6. What is your specialty?	
7. What is your stage of training?	
8. Training region:	Central / West / East/ North/ South / UEA/ Bahrain
9.What is the average working-hours you worked per week over last year?	
10. What is your one-way travel time to work?	Less than 15 minutes/ 15- 30 minutes/ More than 30 minutes
11. Have you been exposed to any form of harassment during your training?	No/ Yes
12. Have you ever been exposed to any Discrimination during your training?	No/ Yes
13. What was the average number of on-call shifts per month in the last 6 months?	
14. Did you drop from your program at any given time since you start your training?	No/ Yes
15. Have you seriously considered quitting specialty at least once in last month?	No/ Yes
16. Have you seriously considered changing your specialty at least once in last month?	No/ Yes
17. How satisfied are you with your current job?	(0 = very little, 6 = very much)
**Health and Daily Habits Information**	
18. What is your weight?	____ kg
19. What is your height?	____ cm
20. Do you have any chronic disease?	No / Yes (If yes, please specify..)
21. Do you have any sleep disorders (e.g., insomnia, sleep apnea, restless leg syndrome, sleepwalking)?	No/ Yes
22. How often do you exercise?	Every day/ 4 times per week/ 3 times per week / 1 time per week/ Rarely
**Maslach Burnout Inventory ™**	
**Perceived Stress Scale**	
**Patient Health Questionnaire-9**	
